# Lectures on Scarlet Fever

**Published:** 1851-07

**Authors:** Caspar Morris

**Affiliations:** Late Lecturer on Practical Medicine in the Philadelphia Medical Institute


MEDICAL EXAMINER,

AND

RECORD OF MEDICAL SCIENCE.

NEW SERIES.—NO. LXXIX.—JULY, 1851.

### ORIGINAL COMMUNICATIONS.

*Lectures on Scarlet Fever.* By Caspar Morris, M. D., late Lecturer on Practical Medicine in the Philadelphia Medical Institute.

### Lecture VI.

Though it is undoubtedly true, that but few diseases are amenable to the influence of purely specific remedies, it is not the less certain, that general principles of treatment must be regulated in their application to each disease, by close observation of the peculiar character and tendencies which mark its own history. No habit can be more pernicious to the student, and less beneficial to the patient, than that so often adopted by superficial thinkers and indolent practitioners, of omitting the consideration of the causes which produce, and circumstances which influence, the progress of any form of disease; and filling the memory with modes of treatment and specific remedies which may be employed empirically, without endeavoring to understand the principles which should determine their adoption, and regulate their application. There is no disease which more fully illustrates this common truth than that now under consideration. It is only from a careful study of its general history, as well as minute observation of the course of symptoms in individual cases, that any suitable idea of treatment can be deduced; and the liability to frequent change in the type of the disease, from simple and sthenic, to irregular and malignant, demanding a corresponding change in the mode of treatment, renders the success of the practitioner equally dependent upon the soundness of his general principles and the accuracy of his particular observation.

Preserving ever certain distinctive peculiarities which prove its identity, yet yielding continually to the influence of those extraneous impressions which govern its development at particular periods, the treatment to be pursued in Scarlet Fever must in like manner be regulated by one leading principle, while it is varied, not only by temporary or local demands, but often in accordance with individual peculiarities. You will find this view of the case has been too little regarded by those writers on the subject whose works are generally consulted by medical students. During some epidemics assuming a sthenic character, in others marked by a strong typhoid tendency; in some, complicated with severe local lesions, while in others, it pursues its course with but little tendency to any particular organ or structure; in some cases, pursuing a course as regular as the progression of the hours; while in others, it only touches at certain points on the confines of the disease, and assumes every conceivable variety of irregularity, it must be at once evident to you that no single remedy or simple plan of treatment could be set forth as appropriate to Scarlet Fever.

There is, however, one idea which should be ever present to your minds, as the regulating principle by which every plan of treatment must be conducted, and which shall place decided limits to the extent to which any course may be followed. Whatever peculiarities may mark the epidemic character, or vary the individual case, scarlet fever in all its varieties, is a disease which has a specific origin, a fixed course, and a certain termination.

There are some diseases in which every hour of continued pain, or disordered function, is a reproach to the skill of the physician; since they are subject to properly applied remedies, and may, by them, be at once subdued. This, on the contrary, is one, the duration of which is fixed and may not be curtailed; and often its tendencies to local destruction may be only moderated, not wholly averted. This fact should never be lost sight of in this and the other kindred diseases which will claim our attention in succession; since he who should attempt to cut short the duration of scarlet fever, would find that it could be done only at the expense of the life of the subject. Even in guarding against local injuries, the same fact should be borne in mind; as, in a large majority of cases, these have a natural tendency to abatement with the decline of the primary symptoms. It was in all probability the observation of these facts which led Sydenham to express his celebrated caution against over anxiety and too vigorous effort; and by the same knowledge we are supplied with power to meet the empty claim of superior success in treatment, put forth with so much vain-glorious boasting by modern empirics. While some cases have a fatal tendency so strong that no skill may avert it, by far the larger proportion of simple and anginose cases need little more than the avoidance of impertinent interference, and the guarding against injurious impressions from without, and they will cure themselves. Let me not be supposed to advocate an indolent indifference, or to assume that no treatment is necessary. There is no disease which demands more watchful care, none is more influenced by skilful scientific practice. I would only remind you that medical science has other remedies than drugs; and caution you against the unscientific resort to empirical perturbative treatment which is certainly more to be dreaded than the morbid impressions of the disease itself. These general principles are applicable to all the various forms assumed by this disease, and must be ever kept present in your mind during the consideration of the various plans of treatment and specific remedies which will be passed in review.

In order to render it more easy for you to embrace the views it is intended to present to you, the same division of the disease will be adopted as was resorted to when describing it. Commencing with the mildest form as affording the type of its character and treatment, we shall be better able afterwards to proceed to the consideration of the graver cases.

In the *simple* form of scarlet fever, where there is merely some vascular excitement, disturbance of the digestive functions, the eruption of the skin and redness of the mucous membrane, without swelling of the throat, nothing more is needed than a mild laxative, followed by a gentle refrigerant and diaphoretic treatment, with the avoidance of fatigue and exposure and the adoption of a careful diet, not only during the four days of febrile action, but until the desquamation is complete. A dose of good calcined magnesia, adapted to the age and other peculiarities of the patient, should be given on the first day of the disease. Lemonade, if the taste of the patient desire it, may be used freely as a drink, and it may be prepared either with simple boiling water, or with barley or rice water; and in either case may be rendered more grateful by the addition of ice. In the case of infants and young children, ice water or gum water is more acceptable than either. As a diaphoretic, the common neutral mixture, prepared with the natural lemon-juice and carbonate of potash, and not with the citric acid of the chemist, is very useful. The addition to this mixture of a small quantity of the spirit of nitrous ether is of service, as it not only increases the tendency to moisture of the surface, but also promotes the secretion of urine, which you remember is liable to be diminished towards the close of the disease. This secretion has also been found to be more acid in this disease than in health, and the use of an alkali was therefore suggested. To meet this indication, the carb. of soda has been much employed, and I can assure you that very many cases have passed through my hands to my entire satisfaction, in which no remedy was employed beyond the weak solution of this salt with the addition of sweet spirits of nitre. I am in the habit of directing the solution of a teaspoonful of the soda in a tumblerful of water, to which one teaspoonful of sweet spirits of nitre is added, and give from one teaspoonful to a dessert-spoonful every hour or two, according to the age of the patient. In these milder cases, the appetite is often but little impaired, and it is therefore important to guard against impropriety in indulgence. I am much in the habit of directing for children the cake known in this city as "lady finger" or Naples' biscuit, which contains nothing but wheat flour with a little sugar and egg; or a thin slice of stale bread toasted quickly; or the biscuit known as "dried rusk," with milk diluted with boiling water sweetened, for the morning and evening meal. Where the fever is very slight, milk-toast without butter may be permitted. It is better that the midday meal should be of the same character if it can be accomplished; and no animal matter, either solid or in the shape of broth or soup, should, on any account, be allowed during the first four days. Where grapes and oranges are accessible, they may be freely used; care being taken that the skins and pulp are not swallowed; and you will find the indulgence of the child in these harmless articles will much abate the desire for other food.

Where the case is more severe and the nervous system more deeply implicated, and we have to contend with muscular twitching, or trembling, or delirium, you will derive the greatest possible benefit from cutaneous ablutions, to be employed conjointly with the treatment first indicated. No slight diversity of opinion with respect to this practice has been expressed by medical writers and practitioners. Under the erroneous impression that some morbid matter was present in the fluids which was to be eliminated by the skin, some have objected to the application of water to the surface, on the ground of its tendency to resist this action; whilst others have feared to resort to it lest it should diminish the cutaneous activity, and produce a transfer of the disease to the nervous centres. Both these views are founded on false notions of the pathology of the disease, and experience has fully proved the apprehensions they suggest are without foundation. Equally great has been the difference of opinion as to the mode of application of water. You must in this, as in all other cases, adapt your application to the object you have in view, and the circumstances of the individual case. In the large majority of instances, the object to be attained is merely the abstraction of heat from the surface, and the consequent diminution of nervous excitement. This may be obtained with the greatest comfort to the patient and least inconvenience to the attendants, by simple sponging with water. It will allay the heat, sooth the tormenting itching of the surface, and reduce the fequency of the pulse. The temperature of the fluid should be adapted to the sensations of the patient. There are great constitutional peculiarities as regards the influence of temperature on the surface. While some persons in health enjoy a high temperature, and find all their functions performed with most comfort during the warm season, others are then languid and oppressed, and long for the return of the cold weather. In the use of baths, a like diversity is found. The languor and depression which in one person will follow the resort to cold bathing, will in another result in equal degree from the employment of the warm bath. Now these same constitutional peculiarities are carried into the hours of sickness. Tepid sponging may always be employed with benefit where the skin is hot and dry, the pulse quick and strong, and the patient restless. Affusion of water at a temperature not less than 96° or 98° of Fahrenheit, will in most similar cases be very beneficial, and this especially in those patients where there is great restlessness and delirium. It allays nervous irritability, and is often followed by refreshing sleep. Currie, of Liverpool, has advocated the use of *cold* affusions in this disease in very strong terms, and he has been followed in this recommendation by other writers. The preponderance of testimony is, however, against it, and it appears to me to be founded on imperfect views of the operation of cold. We are all familiar with the fact, that its primary impression is sedative. This cannot, however, be long maintained without producing serious depression of the vital force. When it is interrupted before this exhaustion is brought about, the result is increased action of the general circulation, and also of the capillaries of the part to which it has been applied. Both these conditions are injurious in scarlet fever: and at the best the cases reported by Dr. Currie prove only that no mischief resulted, since the most he claims is the final abatement of the fever on the fourth day. The remarks of Dr. Chapman on this subject are characterized by that sound discretion, for which he is so remarkable. Especially adapted, in his opinion, to those cases in which the nervous system is most deeply involved, he asserts that he has derived from the application of cold water to the surface, more positive benefit, than from any other remedial measure he has employed. There is one set of cases to which it is peculiarly appropriate; I allude to those in which there is violent delirium, with great heat of skin, very rapid pulse, and extreme restlessness, and not unfrequently convulsions, or at the least violent muscular twitching. Bleeding, whether local or general, is in such cases almost always followed by fatal results. But a cold douche to the head, maintaining the impression by cloths wrung out of iced water, while the whole surface is freely sponged with that fluid at the temperature of the chamber, or but little below it, will do more to procure tranquillity and reduce the nervous excitement, than any other plan that can be adopted. Much important testimony to the value of this treatment, variously modified, might be adduced. By Dr. J. Forsyth Meigs the employment of affusion of water at 96°, containing a small quantity of vinegar, is mentioned with approbation. I have long resorted to baths from 96 to 100°, to which whiskey has been added as a means of tranquillizing young children, and inducing sleep. You will bear in mind that the temperature of the surface in this disease is much exalted above the natural standard of health. When then you resort to the application of water which feels warm to the hand of the person who uses it, it is yet so far below the temperature of the patient that it abstracts heat from the surface, and hence it is that apparently tepid sponging is followed by nearly the same results as those ascribed to more positive cold, and the shock being less it is not so liable to be followed by pernicious reaction. It diminishes the capillary excitement, and maintains a more healthy condition of the cutaneous functions; the interruption to which exerts so baneful an influence on the subsequent course of the disease. The influence of these results on the disease appear sufficiently great to account for the benefit conferred, without adopting the suggestion of Dr. Chapman, that "while mitigating undue excitement by its sedative agency, it also proves more directly the corrective of the effects of the specific virus causing the disease;" to which I draw your attention only for the purpose of exhibiting how high is the estimate he places on this mode of treatment. Dr. Condie is equally decided in the expression of his confidence in it; and Dr. Bell, in his work on baths, asserts "there is no other remedy which unites to any thing like the same extent, efficacy with safety, and immediate pleasurable results, as the cold bath."

If you bear in mind the importance of the functions of the skin, and the great influence the interruption of them must have upon the condition of the circulating fluids, and the nervous centres, you will not think undue prominence is given to this remedial agency. More recently, similar views have led to the adoption of a practice, which, though highly lauded by some of my professional friends who have employed it, when first presented to the mind has no very strong claims to regard. I allude to the employment of inunctions, originally suggested by a German author in the offensive shape of rubbing with the skin of fat bacon. It is rendered less disgusting when modified, as by a recent English writer, who commends it as equally applicable to all febrile diseases, and resorts to an ointment composed of equal parts of suet and lard, which is to be rubbed into the dry skin by vigorous friction. Whatever benefit may accrue from this practice, it can be ascribed only to the soothing influence of the unctuous application to the skin, which acts here as in erysipelas, in which it certainly allays the local irritation, and thus by the removal of one important cause of excitement, may tend to the subduing of the febrile action. How far it may be applicable to the latter stages of the disease, and to those cases in which the sedative influence of cold would be pernicious, I am not prepared to express an opinion; though analogy affords many examples, which might, perhaps, induce some to think favorably of the practice as modified by Mr. Taylor, and to give it a trial, not only in scarlet fever, but in other febrile diseases, with a dry, husky condition of the skin. Nothing can be conceived less likely to promote the restoration of a patient than the German practice as reported in Ranking's Abstract. Not only is the material used for anointing the skin offensive in itself, but directions are given that the clothing should not be changed, "as a clean shirt takes up more of the fatty matter than one already saturated. The rubbing in is to be kept up twice a day for *three weeks*, and once a day *during the fourth*. The patient is, *after this*, to be washed daily with soap and cool water, and then only is the warm hip bath to be commenced." It would require the utmost confidence of success, to reconcile an American mother to such treatment, or induce an American physician to make his daily visits to a chamber so foul. Whatever benefit might accrue from the softening of the cuticle, would be more than balanced by the loathsome effluvia. Nor does the length of time during which the application is to be made, convey an impression at all favorable to the remedial influence of this mode of treatment.

You will thus observe, that in the treatment of the simple form of scarlet fever, I am inclined to adopt a very simple treatment, even in those cases in which some symptoms of a very formidable character present themselves. Emetics recommended by some, may be harmless if employed in the very onset of the disease, when the spontaneous vomiting by which it is often ushered in, would appear to afford a natural indication for their use. If the stomach is known to be loaded with some improper food, or if the effort at vomiting is unsuccessful, a small dose of ipecacuanha may be given to aid in the expulsion of the contents of the stomach. I have never found it necessary to resort to them except in those cases which are ushered in by convulsions as the primary symptom, and which in their onset cannot be known to be scarlet fever, unless some direct communication with the disease, or its presence in the family, should give occasion for suspicion. In either case, the object to be accomplished by the emetic is very simple.

You may remember that when treating of the cause of the disease, I referred to the fact that it is often developed by some agency independent of the specific influence which gives to it its essential character. Whatever disturbs the healthy balance of the system, may thus prove the origin of a case; and among children nothing more frequently acts thus, than impropriety in diet, whether in quantity or quality. Digestion is suspended for the time, either from having been taxed beyond its normal power, or from the depressing influence of the scarlet fever miasm. In either case the presence of the ingestæ in the stomach becomes a source of irritation, which greatly aggravates the danger of the patient. The more promptly this offending matter can be discharged the better; and a dose of ipecacuanha, proportioned to the age of the patient, should be given the moment the power of deglutition is restored. Such cases generally occur in childhood or infancy. The body should be immersed in warm water, while a cold cloth is applied to the head, or cold water poured upon it from a slight elevation. This will almost always suspend the spasmodic action sufficiently long to permit the swallowing of the ipecac. which acts as a simple evacuant.

Purging, beyond the mere evacuation of the bowels as suggested, by magnesia, is positively injurious, and the use of calomel in this disease is, I think so deleterious, that if I were in doubt as regards a case, whether it were scarlet fever or no, I should think the doubt sufficient cause for withholding the use of that remedy, so great is the depressing influence it exerts in its primary impression. There is, in fact, no indication to be met by the use of purgatives; certainly none demanding the resort to mercurials. Neither the symptoms present, nor the results of examination of the bodies of the dead, afford any reason to suspect the presence of those disorders of the viscera, either functional or organic, to the relief of which this remedy is so well adapted. The depressing influence of calomel, and the irritation of the mucous membrane by the other cathartic remedies are both injurious. Bleeding is better endured in the simple form of the disease than in any other. But when the case is not violent it is unnecessary, and if it assumes a deeper grade of severity, my personal experience is very decidedly unfavorable to its employment. I have never seen any good result from it, even in those cases in which the nervous symptoms were most severe, and the vascular excitement the most intense, and I have, alas too frequently! had occasion to mourn over its fatal influence. The impression of cold will, in these cases, be always sufficiently sedative to allay the nervous excitement; and neither analogy nor direct examination of the results on the bodies of those who die, affords any reason to suspect the existence of cerebral inflammation in them. In almost every instance in which I have resorted to depletion, either local or general, in cases of scarlet fever in the primary stages of the disease, no matter how vigorous the patient, or violent the symptoms, the case has had a fatal termination; and in no cases has its evil influence been more manifest than in those in which convulsions or violent delirium, accompanied by sudden shrieks and grinding of the teeth, as in meningitis, would appear to have presented the strongest possible reason for a resort to it. You will find I place an cntirely different estimate on the value of deplētion in the treatment of the sequelæ of this disease.

Permit me to describe to you one case which occurred in what might be properly termed the transition stage of my views of the treatment of this disease. It will illustrate, at the same time, the character of a severe attack of *simple* scarlet fever. The subject was a healthy boy, five years old. He had retired to bed in his usual health. About daylight I was called to see him with convulsions. I found him wholly insensible, with violent spasmodic action of the whole body and all the extremities, pupils contracted, pulse frequent, and skin hot. Though it was impossible to arouse his attention, yet the least disturbance, as in moving him, or administering an enema, produced increased convulsions followed by long continued muscular tremor. On enquiring into the history of the attack, I was informed he had been waked up by sick stomach, and the convulsions had occurred whilst he was in the act of vomiting. Portions of an apple eaten on the previous day were ejected just as he had swallowed it, imperfectly masticated. Supposing the case to be one entirely dependent on indigestion, so frequent among young children, I adopted the treatment appropriate to its relief. A stimulating injection was promptly administered, and the child removed to a warm bath; and so soon as it could be procured, a dose of calomel and ipecacuanha was given during an interval between the convulsions, while mustard cataplasms were applied to the extremities and epigastrium for the purpose of revulsion. At the end of two hours he vomited again, throwing off an additional quantity of undigested apple; soon after which he recovered his consciousness, so far only as to protrude his tongue when solicited to do so. He neither spoke nor manifested consciousness in any other manner. He continued through the day to start much, and manifested no knowledge of the action of the bowels produced by a dose of magnesia given soon after the operation of the emetic. As the convulsions did not return, and the pupils recovered their natural condition, I determined to rely on the counter-irritation of a blister, which was applied to the nape of the neck, and cool cloths kept constantly on the forehead, and to wait for the full effect of the purgative. The pulse continued very rapid throughout the day. The following morning I found him with a vivid eruption on the face and neck, conjunctiva injected, eyes upturned, and pupils contracted, pulse very rapid. The tendons of the wrist twitched incessantly under the finger; he started frequently as though about to spring off the bed, and grated his teeth with much violence.

It was impossible to arouse him; and his pulse was still as frequent as on the previous day. There could be no hesitation in recognizing scarlet fever, even if the tongue had not assumed the peculiar appearance which is so common in that disease. There was, however, no evidence of angina, except some little shrinking on passing the finger beneath the angle of the jaw. The cerebral symptoms were certainly urgent, and appeared to demand the most energetic treatment. In any other disease than scarlet fever, I should not have hesitated to resort to free depletion. Sad experience in several cases, which had then recently passed through my hands to a fatal termination, in which the convulsions and delirium had been even more violent, had led me to determine never again to employ depletion in such cases. I explained to the father my views of the case, told him the result of my observation, assured him of my readiness to meet any other physician of experience and good judgment in consultation on the case, or even, if he desired it, to yield the treatment to another; that if I employed any depletion, it must be to the smallest possible extent. Twelve leeches were applied to the temples, by which but about one ounce and a half of blood was abstracted—and the case was then committed to a mere expectant treatment. The rash was fully developed. There was no affection of the throat, beyond that which was indicated by the tenderness on pressure. The coma gradually subsided from hour to hour, till, on the fifth day, the symptoms all disapeared, and convalescence was fairly established. The favorable result of this case afforded me much encouragement. While in every instance in which active depletion had been employed by me for the relief of the cerebral symptoms, death had ensued; here was a propitious result, though the amount of blood drawn was too small to be entitled to any part of the credit of the case. I was thus confirmed in the opinion, that the cerebral symptoms were purely nervous, and had no connection with inflammation, or even vascular congestion. That the proper indication was only to allay nervous irritation, and to avoid every agent which should prostrate the vital energies. I accordingly from that time adopted a treatment having this object in view, in all similar cases, and have no hesitation in expressing myself entirely satisfied with the result.

I should now not employ even the calomel which was added to the ipecacuanha in this case, under the impression that it was wholly dependant on gastric disturbance,--but should rely on the simple evacuants, to get rid of the ingestæ; following them by the tepid bath or affusion, and the use of simple drinks, with entire rest. You will observe that the *whole train* of morbid phenomena disappeared as the case approached the period at which scarlet fever terminates. I saw it first before daylight on the morning of Tuesday, and on Saturday convalescence was fairly established. To sum up the treatment of simple scarlet fever, I should adopt and recommend that of Dr. Armstrong: "Open the bowels (*not purge*) every day with some mild aperient," (if spontaneously opened, there is no need of medicine). "Keep the patient at rest, between cool clean sheets, on a bed or on a mattress with light clothing, and in a temperature from 56° to 60° F. Sponge the surface with tepid water twice or three times a day, while it is hotter than natural. Admit plenty of fresh air, allow a bland diet, and in two or three days the patient will be well." Above all, do not allow the dread of the name scarlet fever to induce you to adopt a treatment which shall be more injurious than the disease.

(To be continued.)

